# Clinical Value of ¹⁸F-fluorodeoxyglucose Positron Emission Tomography/Computed Tomography in Neuroendocrine Neoplasms: A Case Report and Literature Review

**DOI:** 10.7759/cureus.103838

**Published:** 2026-02-18

**Authors:** David Gutierrez Albenda, Mariana Parra, Ana María Gutiérrez, Ian Taylor, Gabriel Infante

**Affiliations:** 1 Cyclotron-PET/CT Laboratory, Universidad de Costa Rica, San Pedro, CRI; 2 General Medicine, Universidad de Costa Rica, San Pedro, CRI; 3 Medicine, Universidad de Costa Rica, San Pedro, CRI; 4 General Practice, Universidad de Costa Rica, San Pedro, CRI

**Keywords:** 18f-fluorodeoxyglucose, low-grade pancreatic neuroendocrine tumor, neuroendocrine neoplasms, pancreatic neoplasm, pet/ct scan

## Abstract

Neuroendocrine neoplasms (NENs) are rare and heterogeneous tumors in which functional imaging plays a central role in diagnosis, staging, and prognostic stratification. While somatostatin receptor (SSRs)-based imaging remains the standard for well-differentiated tumors, ¹⁸F-fluorodeoxyglucose (¹⁸F-FDG) positron emission tomography/computed tomography (PET/CT) provides complementary information on tumor metabolism and aggressiveness. We present the case of a 52-year-old male diagnosed in 2013 with a low-grade pancreatic neuroendocrine tumor who underwent surgical resection followed by multiple reinterventions due to local progression and mesenteric metastases. Serial SSR PET/CT studies demonstrated high receptor expression in mesenteric lesions. However, subsequent ¹⁸F-FDG-PET/CT revealed a hypermetabolic mesenteric nodal conglomerate, suggesting heterogeneous metabolic behavior and possible tumor dedifferentiation. This case highlights the clinical value of ¹⁸F-FDG-PET/CT in low-grade NENs, particularly for assessing disease progression, by providing prognostic information beyond histological grading alone. ¹⁸F-FDG-PET/CT may identify aggressive tumor components not evident on receptor-based imaging, supporting its role as a complementary tool in selected patients.

## Introduction

Neuroendocrine neoplasms (NENs) are a heterogeneous group of rare malignancies originating from secretory cells of the diffuse neuroendocrine system. They can occur anywhere in the body; up to 75% originate in the gastrointestinal tract and pancreas and are collectively referred to as gastroenteropancreatic NENs (GEP-NENs). The most common sites of development include the small intestine (30.8%), rectum (26.3%), colon (17.6%), pancreas (12.1%), and appendix (5.7%) [1]. The incidence of GEP-NENs is rare, but it has been increasing due to major advances in diagnostic tools and greater medical awareness [2].

NENs are clinically classified as functional (the minority) and non-functional; the latter account for 60-90% and are mostly asymptomatic [3]. Additionally, the World Health Organization classifies them by morphology and aggressiveness: well-differentiated tumors, termed neuroendocrine tumors (NETs), and poorly differentiated tumors, termed neuroendocrine carcinomas (NECs). NETs are slow-growing and are classified according to their Ki-67 proliferation index: grade 1 (G1) with Ki-67 <3%, grade 2 (G2) with 3-20%. NECs, or grade 3 (G3), have an index higher than 20%. The latter reflects a higher rate of cell division and a more aggressive behavior [1-2].

In NETs, ​​the combination of nuclear medicine with radiology, positron emission tomography (PET) with computed tomography (CT), is key to identifying the primary tumor and its extent, staging it, deciding on the therapeutic strategy, monitoring, and prognosis. NENs imaging relies on the overexpression of somatostatin receptors (SSRs) on the cell surface. Radiolabeled somatostatin analogs bind to SSRs on the surface of NET cells. However, SSR-directed imaging is not optimal for evaluating aggressive and poorly differentiated NENs, since these usually lack SSR overexpression [1].

Therefore, ¹⁸F-fluorodeoxyglucose (¹⁸F-FDG) PET/CT (¹⁸F-FDG-PET/CT) has been incorporated into current practice, as it quantifies glucose uptake to assess tumor metabolic activity and predict prognosis, particularly in advanced, moderate- to high-grade tumors [4-5]. ¹⁸F-FDG-PET/CT remains underutilized and controversial in low-grade NETs because these tumors have traditionally been considered metabolically indolent and expected to show low radiotracer uptake. However, its usefulness has recently been demonstrated. In these cases, high ¹⁸F-FDG uptake is associated with a higher risk of early progression, while low uptake is correlated with more indolent tumor behavior [1,6,7]. Thus, although tumor grade is key to selecting the most appropriate radiopharmaceutical, it can evolve over the course of the disease. This highlights an important clinical gap, as histological grading alone may not fully capture the biological heterogeneity or dynamic behavior of NETs. Therefore, it has been proposed that ¹⁸F-FDG-PET/CT could play a broader role across all grades in staging, prognosis, and treatment selection [8].

This report presents the case of a patient with a low-grade pancreatic NET (pNET G1) showing unexpected ¹⁸F-FDG uptake, illustrating how metabolic imaging can provide complementary prognostic and clinical information beyond conventional grading. It also discusses the evolving role of ¹⁸F-FDG-PET/CT in NENs through a focused review of the literature, highlighting its complementary value and limitations relative to SSR-based imaging.

## Case presentation

A 52-year-old male was diagnosed in March 2013 with a pNET G1. At diagnosis, he underwent pancreaticoduodenectomy. Subsequent imaging demonstrated local disease progression, prompting a second surgical intervention. Despite surgical management, disease persistence was documented, and systemic therapy was initiated with sunitinib, followed by everolimus, achieving a partial response before further progression. The patient is currently receiving third-line treatment with long-acting octreotide.

In the year of diagnosis, the patient required surgery for intestinal obstruction caused by a mesenteric mass. Since then, he has experienced multiple local recurrences at the mesenteric root, requiring four surgical reinterventions. Cross-sectional imaging studies (CT and MRI) performed between 2020 and 2023 demonstrated a polylobulated mesenteric lesion with spiculated margins and hypervascular contrast enhancement, progressing in size from 22 × 20 × 29 mm (April 2020) to approximately 65 × 49 × 60 mm (June 2023).

**Figure 1:**
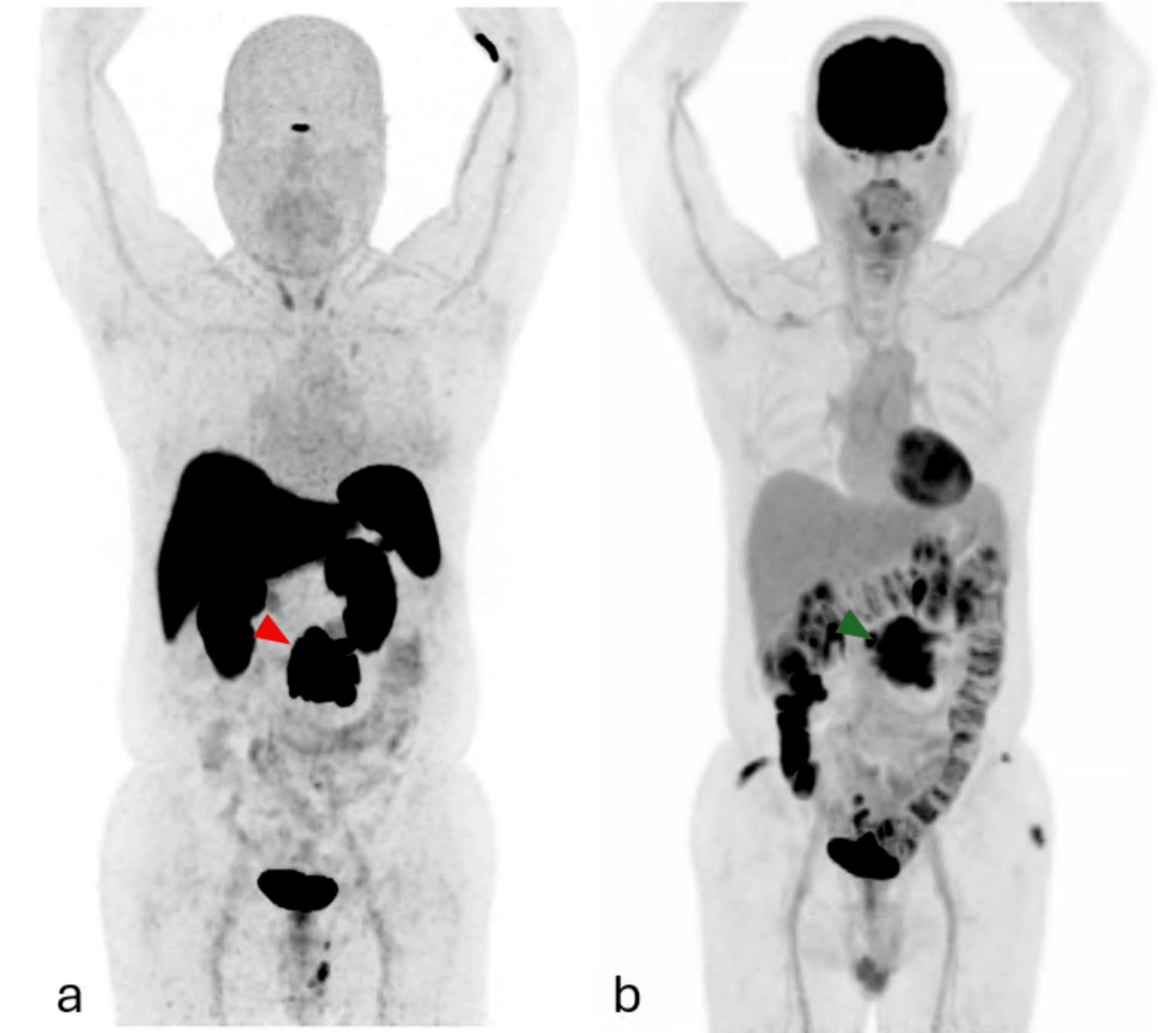
(a) MIP image from SSR PET/CT (December 2024) demonstrating intense radiotracer uptake in a nodal conglomerate in the mesenteric root (red arrowhead). (b) MIP image from ¹⁸F-FDG-PET/CT (May 2025) showing corresponding ¹⁸F-FDG uptake within the same mass (green arrowhead), indicating heterogeneous metabolic behavior MIP: maximum intensity projection, PET/CT: positron emission tomography/computed tomography, ¹⁸F-FDG: ¹⁸F-fluorodeoxyglucose, SSR: somatostatin receptor

**Figure 2:**
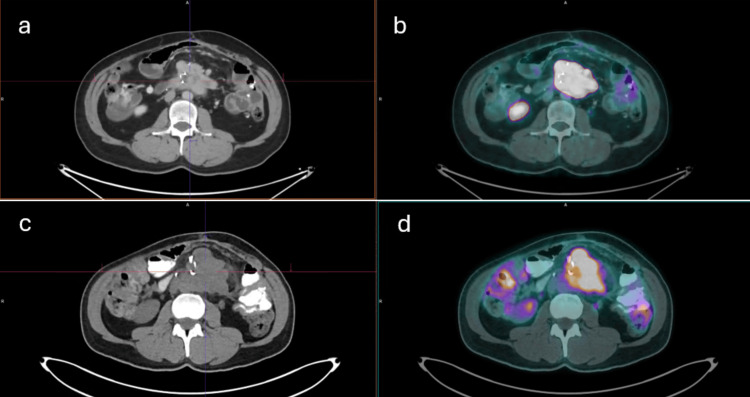
(a, b) Axial CT and axial SSR PET/CT images obtained in December 2024 demonstrating a radiotracer-avid nodal conglomerate in the mesenteric root, consistent with metastatic neuroendocrine disease (SUVmax 70.6). (c, d) Corresponding axial CT and axial ¹⁸F-FDG-PET/CT images obtained in May 2025 demonstrating increased ¹⁸F-FDG uptake within the same mesenteric mass (SUVmax 9.8), reflecting heterogeneous metabolic activity PET/CT: positron emission tomography/computed tomography, ¹⁸F-FDG: ¹⁸F-fluorodeoxyglucose, SSR: somatostatin receptor

Serial PET/CT studies using radiolabeled somatostatin analogs (¹⁸F-AIF-NOTA-octreotide) demonstrated intense SSR overexpression in the mesenteric mass. Uptake was predominantly peripheral, with central areas consistent with degeneration or necrosis. Additionally, a focal area of tracer uptake was identified in the pancreatic tail, corresponding to a slightly hypodense, poorly defined lesion measuring approximately 9 mm. On follow-up imaging in December 2024, the mesenteric conglomerate increased in size (65 × 57 × 70 mm) with the appearance of satellite nodules, while semiquantitative uptake values remained stable, suggesting volumetric progression with internal degenerative changes (Figures 1-2). The pancreatic lesion remained stable in both size and uptake.

At an oncologic evaluation in March 2025, the patient reported weight loss, nausea, abdominal pain, and persistent diarrhea, raising concern for disease progression. These new systemic and gastrointestinal symptoms, in the context of known volumetric progression on anatomical imaging despite stable SSR expression, were considered clinical red flags for possible tumor biological heterogeneity or dedifferentiation in well-differentiated NETs. Specifically, the appearance of systemic symptoms, discordance between anatomical progression and receptor-based functional imaging, increasing tumor burden, and loss of disease control despite ongoing therapy are indications for performing ¹⁸F-FDG-PET/CT, even in low-grade NENs, to assess metabolic aggressiveness and refine prognostic stratification [1,4].

A new PET/CT study was requested. An ⁸F-FDG-PET/CT scan revealed a hypermetabolic nodal conglomerate in the mesenteric root measuring 7.7 × 7.3 × 7.8 cm, with central hypometabolic areas suggestive of necrosis and consistent with neoplastic infiltration. The lesion demonstrated close contact with adjacent small bowel loops and loss of the fat cleavage plane with the abdominal aorta, without definitive evidence of vascular invasion on metabolic imaging. Adjacent fat stranding and satellite nodules with mild ⁸F-FDG uptake were also observed (Figures 1-2). No additional sites of abnormal ⁸F-FDG uptake were identified.

Although SSR PET/CT remains the preferred imaging modality for follow-up of well-differentiated NENs, it was unavailable at the imaging center. Nevertheless, the ⁸F-FDG-PET/CT findings demonstrated significant metabolic activity, suggesting heterogeneous tumor biology and possible dedifferentiation.

## Discussion

NENs are a diverse group of neoplasms ranging from well-differentiated, slow-growing forms to aggressive, high-grade carcinomas [1,2]. Our patient has a pNET G1, which typically exhibits slow growth, good differentiation, and high SSR expression. It is also a nonfunctional pNET, often diagnosed late in the asymptomatic early stages, when it presents with large size, invasion, or metastasis [4].

In NENs, histological evaluation assesses the degree of differentiation and the proliferative index (Ki-67) of tumor cells at the site of collection but does not reflect the tumor's heterogeneity across the body. Furthermore, tumor grade can vary throughout the course of the disease, making functional PET studies essential. This technique provides a comprehensive metabolic overview, useful for initial diagnosis, localization of the primary tumor, assessment of metastatic spread, follow-up, surgical evaluation, evaluation of response to antitumor therapies, and, above all, for prognostic assessment [9]. The integration of PET with CT combines functional information with CT's high anatomical resolution, enabling detection of both the primary tumor and small metastases and aiding therapeutic decision-making [1].

Several studies have suggested that ¹⁸F-FDG-PET/CT may have greater prognostic value than histological classification. To improve prognostic prediction in well-differentiated tumors, a group of authors adjusted the Ki-67 index cutoff from 2% to 5% in a study. Patients with Ki-67 <5% had a lower risk of progression and better survival than those with Ki-67 5%-20%. In analyses of histological and metabolic parameters, ¹⁸F-FDG uptake was the only factor with independent prognostic value [10]. According to Binderup et al. (2021), 31 of 57 (54%) patients classified as G1 died during follow-up; of these, 17 (55%) had tumors with high ¹⁸F-FDG uptake, suggesting that the risk may be underestimated if histological grade alone is considered [9].

¹⁸F-FDG has been routinely used as the primary PET tracer to assess glucose metabolic activity in most cancers. Its use in well-differentiated NENs is controversial because these tumors overexpress SSRs but have low glycolytic activity [4]. However, recent studies demonstrate that even in these tumors, ¹⁸F-FDG can provide relevant prognostic information. In a homogeneous cohort of patients with G1 GEP-NEN, 49% had positive ¹⁸F-FDG uptake, and these patients had significantly shorter progression-free survival than those with negative PET, suggesting that uptake reflects more aggressive disease behavior [5]. In our patient, the mesenteric metastasis demonstrated high ¹⁸F-FDG uptake, which is uncommon in G1 tumors, underscoring the importance of considering ¹⁸F-FDG-PET/CT as an additional imaging modality, even in low-grade NENs. In fact, Asagi et al. [11] demonstrated that ¹⁸F-FDG-PET/CT can alter the therapeutic plan in patients with non-functioning pNETs, ​​as it detects up to 90.5% of metastases, underscoring its usefulness in the staging and prognostic evaluation of this tumor type [12].

In intermediate- and high-grade NENs (G2-G3), ¹⁸F-FDG imaging plays a very important role. These tumors exhibit high glycolytic activity and, conversely, lower SSR expression [1]. In these tumors, higher uptake is associated with poorer prognosis, larger tumor volume, greater aggressiveness, and lower response to SSR-targeted radionuclide therapy (PRRT) [13,14]. Uptake progressively increases from 40% in G1 tumors to 93% in G3 tumors. A maximum standardized uptake value (SUVmax) greater than 10 is associated with poorer response and overall survival [13].

In the imaging evaluation of NENs, radiolabeled somatostatin analogs are also the internationally recommended standard. These images are based on the overexpression of SSRs in well-differentiated NENs, which bind radiolabeled analogs with high affinity [4]. This image allows for whole-body staging, localization of the primary tumor, and detection of metastases, especially useful in well-differentiated NETs, ​​where it achieves sensitivities of 91-100%. In contrast, in high-grade NENs, SSR expression decreases significantly; therefore, ¹⁸F-FDG-PET/CT is more useful for assessing aggressiveness [1,4,15].

Dual imaging with radiolabeled somatostatin analogs and ¹⁸F-FDG can therefore be used as a valuable tool to assess tumor heterogeneity and optimize prognostic and therapeutic stratification in NENs [4]. For this purpose, the NETPET score was developed, which combines both pieces of information into a single imaging biomarker [4,13]. This classification (P1-P5) distinguishes tumors from highly differentiated with somatostatin uptake alone to dedifferentiated tumors with ¹⁸F-FDG uptake alone, correlating with histological grade and prognosis [4,16]. In several studies, the dual approach modified therapeutic decisions in up to 40% of patients, guiding treatment choices between surgery, PRRT, and systemic treatment [13,14].

The liver, mesentery, and peritoneum are common sites of metastasis in GEP NENs. Lymph node metastases at the root of the mesentery often cause bowel retraction, resulting in intestinal obstruction, as in our patient's case. In pNETs, ​​dissemination to the mesentery is uncommon compared to small bowel NETs, ​​which prefer this location. In our patient, despite being a G1 pNET, the mesenteric metastasis had high ¹⁸F-FDG uptake (SUVmax 9.8), which is not an expected pattern. This suggests metabolic heterogeneity within the tumor, underscoring the value of this study in identifying lesions with higher prognostic risk that may be missed by SSR-only studies [17].

Importantly, ¹⁸F-FDG-PET/CT provided complementary quantitative and functional information that was not apparent in prior SSR PET/CT studies, in which uptake values remained stable despite a progressive increase in lesion size. The demonstration of focal high ¹⁸F-FDG avidity within a growing mesenteric mass supported the suspicion of biologically more aggressive tumor components. It contributed to a multidisciplinary reassessment of disease behavior and prognosis, reinforcing the need for closer clinical and imaging surveillance [10].

Follow-up of patients with PNETs should include clinical and biochemical assessments, as well as conventional and functional imaging with PET/CT. International guidelines recommend adjusting testing frequency based on tumor grade and Ki-67 index: every 6-12 months for G1-G2 NETs with Ki-67 <5%, and every 2-3 months for G3 tumors with Ki-67 >5%. In patients operated on with complete resection, without metastases, monitoring should be carried out for at least 10 years, and in GEP NETs, ​​it is recommended for life. Selection of the method and frequency should be based on the physician's judgment, taking into account the patient's age, the presence of metastases, and the disease trajectory [1].

This report has several limitations that should be acknowledged. As a single-case report, the findings cannot be generalized to all patients with low-grade NENs. Additionally, because our institution serves as a referral center for diagnostic imaging and is not directly involved in patient management, histopathologic confirmation following ¹⁸F-FDG-PET/CT was unavailable. The metabolic imaging findings were interpreted in the context of clinical evolution and serial imaging rather than by tissue sampling. Nevertheless, the case illustrates a clinically relevant scenario increasingly recognized in the literature, in which ¹⁸F-FDG-PET/CT provides prognostic information beyond conventional grading and receptor-based imaging, supporting its selective use in well-differentiated NENs with atypical or progressive behavior.

## Conclusions

NENs represent a heterogeneous group of rare tumors with variable biological behavior. While SSR PET/CT remains the imaging modality of choice for well-differentiated NENs, ¹⁸F-FDG-PET/CT provides valuable complementary information, particularly for disease progression, metabolic heterogeneity, or suspected dedifferentiation. ¹⁸F-FDG uptake correlates with tumor aggressiveness and prognosis and may identify high-risk disease not apparent on receptor-based imaging alone. Therefore, ¹⁸F-FDG-PET/CT should be considered a useful adjunct to the comprehensive evaluation of selected patients with low-grade NENs.
